# 

**Published:** 1858-12

**Authors:** 


TWELFTH VOLUME

OF THE

### DENTAL REGISTER OF THE WEST.

EDITED BY

J. TAFT and G. WATT.

TERMS: . . . . . . . . . $3 PER ANNUM, IN ADVANCE.

**Advertisements Inserted as follows:**

One page, per annum,......................................................$25 00
One-half page, per annum.................................................. 15 00
Quarter page, per annum,.................................................. 10 00

Shorter periods than one year, by special arrangement.

☞Contributions to the pages of the *Register* respectfully solicited.

Exchanges and Correspondents will please address "Dental Register," or "J. Taft, Cincinnati, Ohio."

